# Thin Cartilage Cap May Be Related to the Spontaneous Regression in Pediatric Patients with Osteochondroma

**DOI:** 10.3390/curroncol29120777

**Published:** 2022-12-15

**Authors:** Ryohei Adachi, Tomoki Nakamura, Kunihiro Asanuma, Tomohito Hagi, Teruya Uchiyama, Akihiro Sudo

**Affiliations:** Department of Orthopaedic Surgery, Mie University Graduate School of Medicine, Tsu 514-8507, Japan

**Keywords:** spontaneous regression, osteochondroma, cartilage cap

## Abstract

Background: The spontaneous regression of osteochondromas is rare, and only a few cases have been reported. Furthermore, the precise mechanism underlying spontaneous regression is unknown. This study aimed to examine the radiological findings of osteochondromas that had spontaneous regression and to identify potential indicators of this uncommon phenomenon in skeletally immature patients with osteochondromas. Methods: We included 28 patients (15 males and 13 females) who met the eligibility criteria between 2002 and 2019. The mean age at initial diagnosis was 9.7 years old (2–16 years). The mean follow-up period was 6.4 years (3–16 years). Results: Of the 28 patients, 10 (35.7%) had osteochondroma resolution. The osteochondroma resolved in one patient and regressed in nine. Tumor shrinkage is related to the thickness of the cartilage cap. The thickness of the cartilage cap did not correlate with age. Conclusions: Tumor shrinkage is associated with a thinner cartilage cap on magnetic resonance imaging. The thickness of the cartilage cap may be an important predictor of spontaneous regression in pediatric patients with osteochondroma.

## 1. Introduction

Osteochondroma is a cartilage-covered bone tumor that accounts for up to 50% of all benign bone tumors and 35% of all primary benign bone tumors in children [1,2]. Endochondral ossification of proliferated cartilage is required for tumor growth [3]. The lesion may develop during childhood but does not develop after puberty [4,5]. Approximately 85% of osteochondromas are nonhereditary solitary lesions [5,6]. Malignant transformation, which usually occurs within the cartilage cap and leads to the development of secondary chondrosarcomas, is the most severe complication of osteochondroma [7]. It is estimated that malignant transformation occurs in approximately 1% of solitary osteochondromas [8,9]. However, the spontaneous regression of osteochondroma is poorly understood. Paling [10] describes the first case of spontaneously vanishing osteochondroma, and since then, a few spontaneous regressions of osteochondromas have been reported [11]. Although the exact mechanism of spontaneous regression is unknown, osteochondroma regression can occur either before or after growth plate closure via gradual absorption of the tumor stalk [12]. This study aimed to examine the radiological findings of osteochondromas that had spontaneously regressed and identify the possible indicators of this uncommon phenomenon in skeletally immature patients.

## 2. Materials and Methods

**Figure 1:**
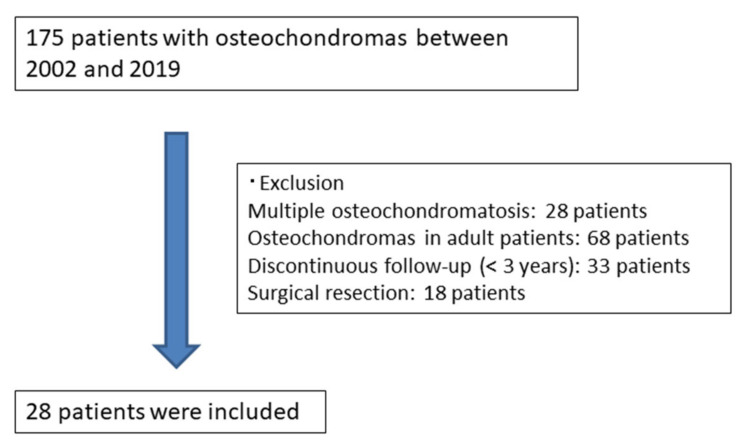
The patients’ demographics.

Between 2002 and 2019, 175 patients with osteochondromas were enrolled in the database of our institution. Of the 175 patients, we included 28 (15 males and 13 females) who met the inclusion criteria (Figure 1). We excluded 68 skeletally mature patients, 28 patients with multiple osteochondromas, and 33 patients lost to follow-up. Furthermore, 18 patients were excluded because they underwent surgical resection within 3 years of their initial presentation; the surgeries were performed because of disturbances in the excursion, cosmesis, and severe pain. The mean age at initial diagnosis was 9.7 years old (2–16 years). The mean follow-up period was 6.4 years (range, 3–16 years). The minimum follow-up duration was 3 years. Of the 28 enrolled patients, eleven cases of osteochondromas regression were observed in the tibia (49%), six in the femur (21%), four in the humerus (14%), and seven in other bones (25%).

### 2.1. Assessment of Osteochondroma

Aiba et al. assessed tumor shrinkage on radiography using three tumor dimensions (base, stalk, and height) [12]. Tumor shrinkage was determined based on these assessments (Table 1):

Vanished: More than 70% decrease in any tumor dimension, either at the tumor base, stalk, or in relation to tumor height relative to the tumor at maximum size.

Regressed: A decrease in any tumor dimensions, including the base, stalk, and tumor height of >30% but <70% relative to the tumor at maximal size.

They classified the mode of tumor regression according to the three theories that were used in this study (Table 1) [12]:

The tumor’s height was used to determine incorporation, which took transverse enlargement of the bone into account. If the decrease at the bone base was >30% and there was an equivalent increase in the distance between the bone axis and the base of the tumor, we referred to the tumor as incorporated.

The tumor stalk was used to assess absorption. If a decrease >30% was observed in the tumor stalk, it was referred to as absorbed.

Fracture: The regression mode was a fracture when it was confirmed during the follow-up period and remained at the tumor base.

### 2.2. Statistical Analysis

The Mann–Whitney U test was used for quantitative data and the chi-square test for qualitative data. *p* < 0.05 was considered to be statistically significant. EZR graphical user interface (Saitama Medical Center, Jichi Medical University, Saitama, Japan) was used for all statistical analysis for R (R Foundation for Statistical Computing, Vienna, Austria) a modified version of the R computer. This is designed to add statistical functions frequently used in biostatistics.

## 3. Results

At the time of initial presentation, the mean thickness of the cartilage cap in all 28 patients was 3.0 mm (1.0–7.7 mm) upon MRI examination. Seven patients had pedunculated tumors, while twenty-one patients had sessile tumors. Of the 28 patients, 10 (35.7%) had osteochondroma regression. Osteochondromas resolved in one patient and regressed in nine (Table 2). The mean size of the cartilage cap in the 10 patients was 2.29 mm (1–4.57 mm).

Tumor shrinkage was observed in seven of the twenty-one patients with sessile tumors (33%), and three of the seven patients with pedunculated-type tumors (43%). Four cases of regression via incorporation (57.1%) and three cases via absorption (42.9%) occurred in seven cases with sessile tumors. Regression occurred via absorption (75%) in three cases with pedunculated-type tumors and via fracture (25%). In the 10 patients, the mean size of the cartilage cap was 2.29 mm (1–4.57 mm). Osteochondromas were stable in nine (32% of patients) and progressed in nine (32%). Table 3 shows that tumor shrinkage was related to cartilage cap thickness (*p* = 0.038, Mann–Whitney U test). Regression was unrelated to sex, age, tumor site, or tumor shape.

The relationship between the cartilage cap thickness and the clinical variables is presented in Table 4. The cartilage cap thickness was not associated with age, sex, tumor site, or tumor shape. This result suggests that cartilage cap thickness may be an important predictor of spontaneous regression in pediatric patients with osteochondroma.

### 3.1. Case Presentations

#### 3.1.1. Case 1

**Figure 2:**
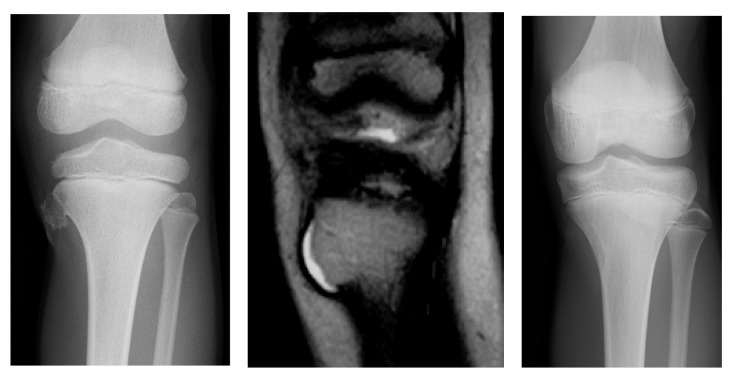
A radiograph revealing a pedunculated osteochondroma at the proximal tibia (left). The thickness of the cartilage cap is 2.83 mm upon MRI examination (middle). After 6 years, the tumor vanished, as depicted by a radiograph (right).

A 6-year-old boy presented with a painful mass in the left proximal tibia. A radiograph revealed a pedunculated osteochondroma measuring 11 × 11 × 10 mm in the proximal tibia. There were no hereditary or multiple lesions found. There had been no history of trauma or vehicular accidents. MRI revealed that the thickness of the cartilage cap was 2.83 mm at the time of the initial diagnosis (Figure 2). A conservative observation was performed. After 1 month, the patient experienced rapid pain relief. After 6 years, the patient was still asymptomatic, and the bony mass was not palpable on physical examination. A recent radiograph demonstrated a vanishing lesion.

#### 3.1.2. Case 2

**Figure 3:**
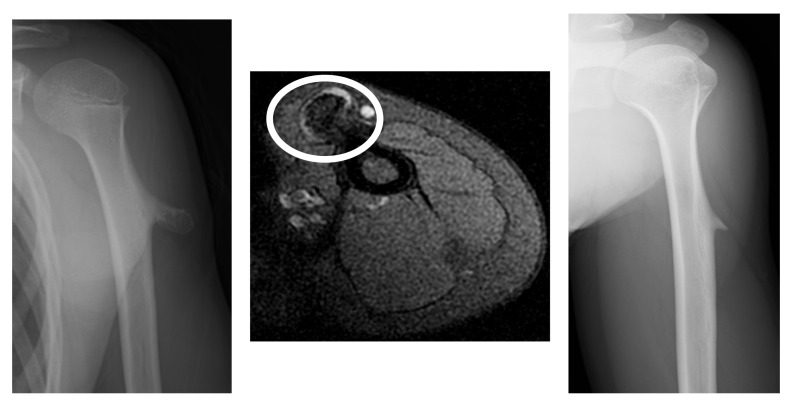
Radiographs revealing a pedunculated-type osteochondroma of the proximal humerus (left). At initial diagnosis, the thickness of the cartilage cap is 2.25 mm (circle) on MRI (middle). Two years later, regression of osteochondroma was observed (right).

A 10-year-old girl presented with a painless lump on her left humerus. There was no history of trauma or other symptoms, and there was no evidence of hereditary or multiple lesions. Radiographs revealed a pedunculated humeral osteochondroma. MRI revealed that the thickness of the cartilage cap was 2.25 mm at the time of the initial diagnosis (Figure 3). A conservative observation was performed. Osteochondroma regression was observed 2 years later in the left humerus.

## 4. Discussion

In this study, we found that 10 of the 28 patients who were followed up for >3 years had osteochondroma regression. The osteochondroma vanished in one patient and regressed in nine. At the time of initial presentation, the thickness of the cartilage cap on the MRI was related to tumor shrinkage. Spontaneous regression of solitary osteochondromas is a rare phenomenon [6,11]. The precise mechanism of spontaneous osteochondroma resolution is unknown. In this study, we found that the age at initial presentation, sex, bone site (long or flat bone), and type of osteochondroma were not related to spontaneous resolution. We found that the cartilage cap at the time of initial diagnosis may be a predictor of future spontaneous regression. A cartilage cap thickness >3 cm in children or 2 cm in adults is a sign of malignant transformation [13,14,15]. The maximum diameter of the cartilage cap in this study was 7.7 mm because we performed surgical resection in children if the cartilage cap thickness was >2 cm. Florez et al. reported that two of the 113 solitary osteochondromas treated between 1970 and 2002 transformed into secondary chondrosarcomas [9]. They concluded that the presence of pain and the growth of osteochondroma in older patients indicated a possible malignancy. However, there are no data on the relationship between cartilage cap thickness and spontaneous regression.

Durán-Serrano et al. reviewed 32 patients with spontaneously regressed solitary osteochondroma [11]. They found fourteen cases of the distal femur and five cases of proximal humeral osteochondroma regressions likely to occur in the proximal tibia. This could be because of the high prevalence of osteochondromas in these areas. In this study, the humerus (30%), femur (30%), tibia (30%), and others (10%) all had similar regions where spontaneous regression of osteochondromas occurred. They recommend that MRI should be limited if other diagnoses, such as rapidly growing lesions and neurovascular compromise, are likely. However, we recommend using MRI at the initial presentation because it can reveal specific characteristics of the osteochondroma’s cartilaginous cap. However, if general anesthesia is required due to age, an MRI examination can be postponed, and an annual radiographic follow-up could be an option. Hoshi et al. proposed that if the osteochondroma is asymptomatic and has typical radiographic findings, careful observation may be acceptable, and occasionally spontaneous regression may be observed [16].

Aiba et al. reported that tumor morphology is related to tumor regression [12], and the shrinkage of pedunculated tumors is caused mainly by absorption or fractures. However, shrinkage of sessile tumors is caused mainly by incorporation or absorption. In this study, tumor shrinkage occurred in seven of twenty-one patients (33%) with sessile tumors and three of seven patients (43%) with pedunculated tumors. The rate of spontaneous regression did not differ significantly between the two types. Shrinkage occurred via absorption in three patients and fracture in one patient in four cases of pedunculated tumors. Shrinkage occurred via incorporation in four patients and absorption in three patients in seven cases of sessile tumors. They examined the relationship between cartilage cap size and osteochondroma regression. However, no statistically significant finding was found in the study’s collection of magnetic resonance imaging. The average size of the cartilage cap at initial diagnosis in the case of a shrinking tumor in the cohort was 2.3 mm (range: 1.3–3.3 mm). During the final follow-up period, all such cases had regressed, almost vanishing. We support their results by demonstrating a significant relationship between the size of the cartilage cap and osteochondroma regression.

Occasionally, spontaneous regression of multiple osteochondromatoses has been reported [17,18]. Because of the small number of patients with multiple osteochondromatoses who could be radiologically followed up for all lesions, we were unable to include these patients. Mordenti et al. reported the natural history of 158 pediatric patients with osteochondromatosis and found that 46.2% of patients had disease progression, while spontaneous regression occurred in five patients (3.2%) [17].

Our study has a few limitations. First, an MRI was not performed when tumor shrinkage was observed. However, the precise timing of the appearance of osteochondroma is unknown. In addition, the exact timing of the regression was unknown. Therefore, we believe that the thickness of the cartilage cap at the initial presentation may be sufficient for predicting tumor shrinkage. Second, there was a lack of histopathological confirmation. Third, a small number of patients were included in the study. Further large-scale studies are required to validate our findings.

## 5. Conclusions

We examined 28 pediatric patients with solitary osteochondromas. Of the 28 patients, osteochondroma regression was observed in 10. MRI revealed that a thinner cartilage cap is associated with tumor shrinkage. Cartilage cap thickness may be an important predictor of spontaneous regression in pediatric patients with osteochondroma.

## Figures and Tables

**Table 1:** Definition of tumor regression.

Tumor Regression	
Vanished	>70% decrease from baseline
Regressed	A 30–70% decrease from baseline
**Assessment Method for Mode of Tumor Regression**	
Incorporation	The height of the tumor
Absorption	The stalk of the tumor
Fracture	History of fracture

**Table 2:** The characteristics of the patients with spontaneous regression.

Age (Years)	Sex	Thickness of Cartilage Cap (mm)	Type	Site	Age at Shrinkage (Years)
2	Female	3.84	Sessile	Tibia	8
7	Male	2.83	Pedunculated	Tibia	11
9	Male	4.57	Sessile	Humerus	13
9	Female	1	Sessile	Femur	15
8	Male	1	Sessile	Tibia	9
3	Male	1.61	Sessile	Femur	12
10	Female	2.25	Pedunculated	Humerus	14
10	Male	1.91	Pedunculated	Femur	11
11	Female	1.37	Sessile	Humerus	12
7	Female	1	Sessile	phalanx	9

**Table 3:** The variables for predicting tumor shrinkage.

Variables	Category	Regression	Stable or Progression	*p*-Value
Sex	Male	5	10	1.0
	Female	5	8	
Age	Mean (range)	9 (2–12)	10 (2–16)	0.346
Tumor site	Long bone	9	12	0.362
	Flat bone	1	6	
Shape of tumor	Pedunculated	3	4	0.674
	Sessile	7	14	
Cartilage cap	Mean (range)	2.29 mm (1.0–4.57)	3.65 mm (1.6–7.65)	0.038

**Table 4:** The relationship between cartilage cap and clinical variables.

Clinical Variables	Category	Mean Cartilage Cap	*p*-Value
Sex	Male	2.86	0.351
	Female	2.09	
Age	10>	2.93	0.162
	10≤	2.01	
Tumor site	Long bone	2.7	0.362
	Flat bone	1.35	
Shape of tumor	Pedunculated	3.24	0.674
	Sessile	2.26	

## Data Availability

The data presented in this study are available on request from the corresponding author.
